# Analysis of the Antiproliferative Effects of Curcumin and Nanocurcumin in MDA-MB231 as a Breast Cancer Cell Line

**Published:** 2016

**Authors:** Mohammad Hossein Khosropanah, Amin Dinarvand, Afsaneh Nezhadhosseini, Alireza Haghighi, Sima Hashemi, Fereidon Nirouzad, Sepideh Khatamsaz, Maliheh Entezari, Mehrdad Hashemi, Hossein Dehghani

**Affiliations:** a *Department of Medicine, Dezful University of Medical Sciences, Dezful, Iran. *; b *Department of Medicine,Tehran medical Sciences Branch,IslamicAzad University, Tehran, Iran.*; c *Department of Laboratory Sciences,Tehran Medical Sciences Branch, Islamic Azad University, Tehran, Iran.*

**Keywords:** Anticancer activity, Curcumin, Nanocurcumin, MTT assay, Human breast adenocarcinoma

## Abstract

Cancer is one of the main causes of mortality in the world which appears by the effect of enviromental physico-chemical mutagen and carcinogen agents. The identification of new cytotoxic drug with low sid effects on immune system has developed as important area in new studies of immunopharmacology. Curcumin is a natural polyphenol with anti-oxidative, anti-inflammatory and anti-cancer properties. Its therapeutic potential is substantially hindered by the rather low water solubility and bioavailability, hence the need for suitable carriers. In this report we employed nanogel-based nanoparticle approach to improve upon its effectiveness. Myristic acid-chitosan (MA-chitosan) nanogels were prepared by the technique of self-assembly. Curcumin was loaded into the nanogels. The surface morphology of the prepared nanoparticles was determined using SEM and TEM. The other objective of this study was to examine the in vitro cytotoxic activity of cell death of curcumin and nanocurcumin on human breast adenocarcinoma cell line (MDA-MB231). Cytotoxicity and viability of curcumin and nanocurcumin were assessed by 3-(4,5-dimethylthiazol-2-yl)-2,5-diphenyltetrazolium bromide (MTT) and dye exclusion assay.

Transmission electron microscopy confirmed the particle diameter was between 150 to 200 nm. Proliferation of MDA-MB231 cells was significantly inhibited by curcumin and nanocurcumin in a concentration-dependent manner in defined times. There were significant differences in IC50 curcumin and nanocurcumin. curcumin -loaded nanoparticles proved more effective compared to TQ solution. The high drug-targeting potential and efficiency demonstrates the significant role of the anticancer properties of curcumin -loaded nanoparticles.

## Introduction

In recent years, the morbidity and mortality of cancer still reaches a high plateau and is a major public health problem world-wide. (1, 2)

Cancer is the major cause of human’s death because of high incidence and mortality.The identification of new cytotoxic drug with low sid effects on immune system has developed as important area in new studies of immunopharmacology. (3)

Many studies report that a high diet in fruits and vegetables lowers the incidence of cancer. (4, 5) 

 Curcumin is a multi-functional and pharmacologically safe natural agent. Chemically it is a low-molecular- weight polyphenol derivative, extracted from rhizomes of *Curcuma *species. Used as a food additive for centuries, it has been recently demonstrated that curcumin is highly pleiotropic, interacting physically with diverse molecular targets, which includes transcription factors, growth factors and their receptors, cytokines, enzymes, and genes regulating cell proliferation and apoptosis (6). Curcumin possess potent antiinflammatory, antitumor and antioxidative (free radical scavenging activity) properties. Preclinical data shows that curcumin inhibits the formation of tumors in animal models of carcinogenesis, induce apoptosis in cancer cells of different tissues or organs, such as colon, breast, prostate and lung, acting on a variety of signal transduction pathways and molecular targets involved in the development of cancer (7). The ability of curcumin to induce apoptosis in cancer cells, without cytotoxic effects on the healthy ones, is suggestive of a relevant anticancer potential. For instance, curcumin leads to apoptosis in scleroderma lung fibroblasts without affecting normal lung fibroblasts (8, 9). Despite all these promising characteristics, a major problem with curcumin is the very low solubility in aqueous solutions, which limits bioavailability and clinical efficacy. Interest in the development of nanocarriers for curcumin therapy is emerging.

Drug overdose remains a common clinical problem across the globe, occurring both inadvertently and intentionally. Ideally, an antidote is provided, but specific antidotes are frequently not available. Consequently, a broad range of non-specific therapies have been developed to approach this problem, ranging from cathartics to more invasive and sophisticated approaches Nanoparticles have been developed for scavenging various drugs; indeed (10). Nanogels, sub-micron hydrogel nanoparticles, have highly desirable properties that may make them particularly suitable for such applications. Like hydrogels, nanogels have a threedimensional, internally crosslinked microstructure, swell in aqueous solvents to provide free volume for non-specific sorption, and can shrink and swell according to changes in the gel environment. Like nanoparticles, nanogels are injectable, have extremely high specific surface areas available for interaction with chemicals in the gel environment, and respond much faster to environmental stimuli. Based on these properties, nanogels have already attracted significant interest as sensors, rheological modifiers, optical devices, mechanical actuators, diagnostics supports, and drugdelivery vehicles, among other applications (11).

 In this study, we show that self-assembled nanogels obtained from Myristic acid - Chitosan are effective curcumin nanocarriers . The aims of the current study were: 1) the preparation of MA-chitosan nanogels loaded with curcumin and 2) Comparison the effects of curcumin and nano curcumin on the cytotoxicity of human breast adenocarcinoma cell line.

## Experimental

***Chemicals***

Curcumin ((1E, 6E)-1,7-bis (4-hydroxy- 3-methoxyphenyl) -1,6- heptadiene-3,5-dione) was purchased from Sigma Chemical Co. (St. Louis, MO. USA).

*Preparation of the nanogels.*

**Figure 1:**
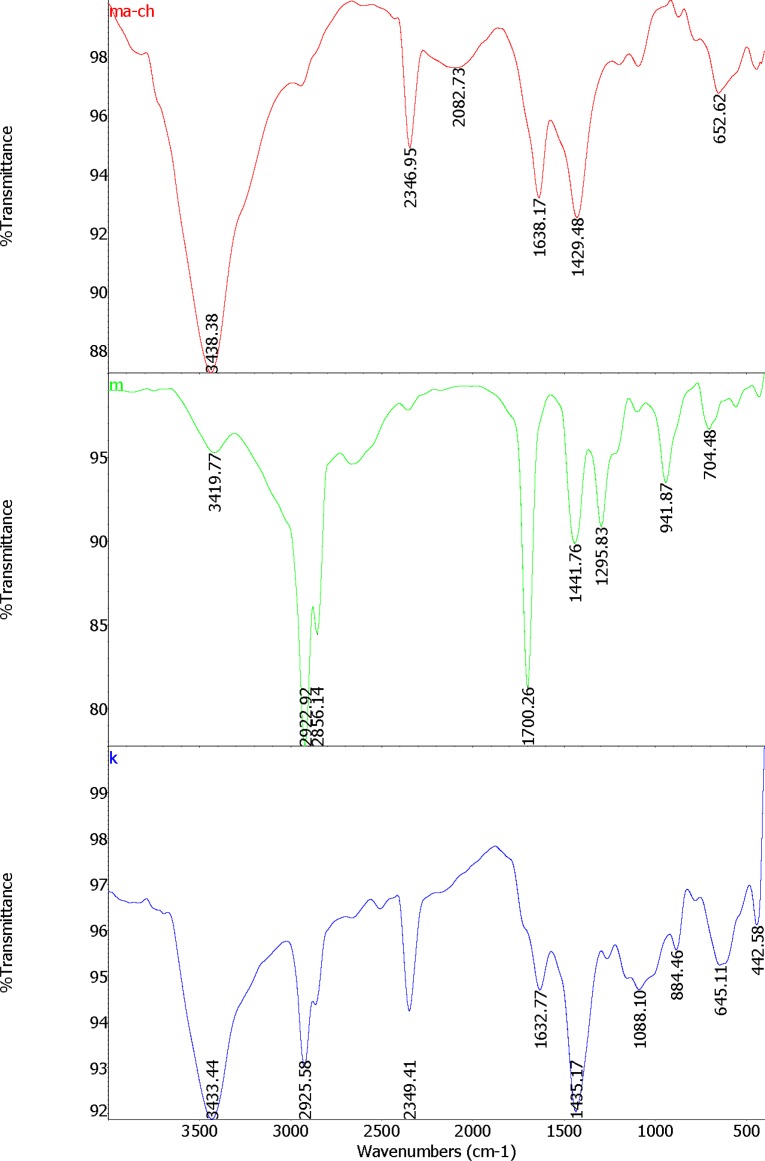
FTIR spectrum of MA-chitosan nanogels (ma-ch), Myristic acid (m) and chitosan (k).

Nanogels were prepared by the technique of self-assembly with the method of Chen, *et al*. (12) with some modifications. Myristic acid (MA) solution was prepared by dissolving 228 mg MA (Sigma Aldrich ≥ 99%, Mw = 228.37 g/mol) in 23 mL Dimethyl sulfoxide (DMSO, Sigma Aldrich, ≥ 99.9%, Mw = 78.13 g/mol). Chitosan (0.5 g) (Sigma Aldrich, medium molecular weight of 340 g/mol) was dissolved in 1% acetic acid solution (100 mL). Polymer complexes of MA and chitosan was prepared by adding 1-ethyl-3-(3-dimethylaminopropyl)-carbodiimide (EDC) (Sigma Aldrich, Mw = 155.24 g/mol, d = 0.814 g/mL) cross linker. EDC was dissolved in methanol (126 µL/15 mL); then added to the MA solution. Subsequently, chitosan solution was added drop wisely to the MA solution while stirring under the hood at room temperature. MA was coupled to chitosan by the formation of amid linkages through the EDC-mediated reaction. The weight ratio of chitosan to MA and EDC were 3:1:1. The molecule oriented itself to exposed hydrophilic regions to the water and the hydrophobic regions assembled in the core; by this process micelle like nanogels are prepared. FTIR spectra of the formed nanogels were studied as represented in Figure 1. Dried nanogels were dissolved in 1% acetic acid solution and kept in refrigerator at -4 ºC until required for the tests. Dionized water was applied for preparing all solutions.

*Loading C* *urcumin*

Curcumin was loaded into the MA-chitosan nanogels. A variable ratio of curcumin was added to the nanogels while sonicating in water bath (Powersonic S10) with high-power ultrasound at room temperature for 30 min. Curcumin -loaded nanogels (CLNs) were characterized by Malvern Zetasizer (Malvern Instruments Ltd., United Kingdom) to determine median size of nanogels and size distribution when loaded with different amount of the curcumin. Scanning and Transmission Electron Microscopy (SEM and TEM) was preformed to observe CLNs morphology, shape, size and core and shell structures. For preparing SEM photographs, one drop of CLN was dropped onto studs and gold sputtered using an auto sputter coater (BIORAD Polaron Division), then analyzed with Hitachi Field Emission Scanning Electron Microscope (FE-SEM) S-4160 (Japan) at 15 kV acceleration voltage. TEM was performed with the method of Liu et al. by using Transmission Electron Microscope (Zeiss EM 10 C TEM-GmbH) at an accelerating voltage of 80 Kv (13). The increasing amount of the curcumin led to an increase in the particles sizes with irregular shape (Figure 1). 

*Cell culture and* *curcumin* *treatment*

MDA-MB231 cell line was obtained from the National Cell Bank of Iran (NCBI). The cells were cultured in RPMI-1640 containing 10 % FBS, 2 mM glutamine, antibiotics (penicillin G, 60 mg/L; streptomycin, 100 mg/L; amphotericin B, 50 μL/L) under a humid atmosphere (37 °C, 5 % CO2, 95 % air). 

Upon reaching appropriate confluence, the cells were passaged. The cells were incubated with 0,10,25,50,100, and 200µg/mL concentrations of curcumin and nanocurcumin for defined times. . The cells were treated with MA-chitosan nanogels at the same dose of that used in the maximum dose (200 microgram/mL) group too.

*Determination of cell viability*

The assay detects the reduction of MTT (3-(4,5-dimethylthiazolyl)-2,5-diphenyl-tetrazolium bromide) (Sigma) (a colorimetric technique) by mitochondrial dehydrogenase to blue formazan product, which reflects the normal function of mitochondria and hence the measurement of cytotoxicity celland viability.

Briefly, l0^4^ cells/well were treated with various concentrations of curcumin and nanocurcumin. After 24 h incubation the cells were washed twice with phosphate buffered saline (PBS) and MTT (0.5 mg/mL PBS) was added to each well and incubated at 37°C for 3h. The formazan crystals that formed were dissolved by adding dimethyl sulfoxide (100 μL/well), and the absorbance was read at 570 nm using a microplate scanning spectrophotometer (ELISA reader, Organon Teknika, Netherlands). Toxicity level was calculated by the following formula:

$\mathrm{Cytotoxicty}=1-\frac{\mathrm{mean ab}\mathrm{sorbance of toxicant}}{\mathrm{mean absorbance of negative control}}\times 100$

(1)

Viability % = 100 - Cytotoxicity %

To diminish test error level, MTT strain was added to some wells without cells and along with other wells, absorbance level was read and ultimately subtracted from whole the absorbance.

*Statistical analysis*

Statistical significances of difference throughout this study were calculated using a Student^,^s t-test and by one-way variance analysis.

## Results

*Preparation and validation of nanogel*

A FTIR-Spectrum validated proper formation of the MA-chitosan nanogels (Figure1).

***Surface morphology (TEM and SEM study)***

**Figure 2:**
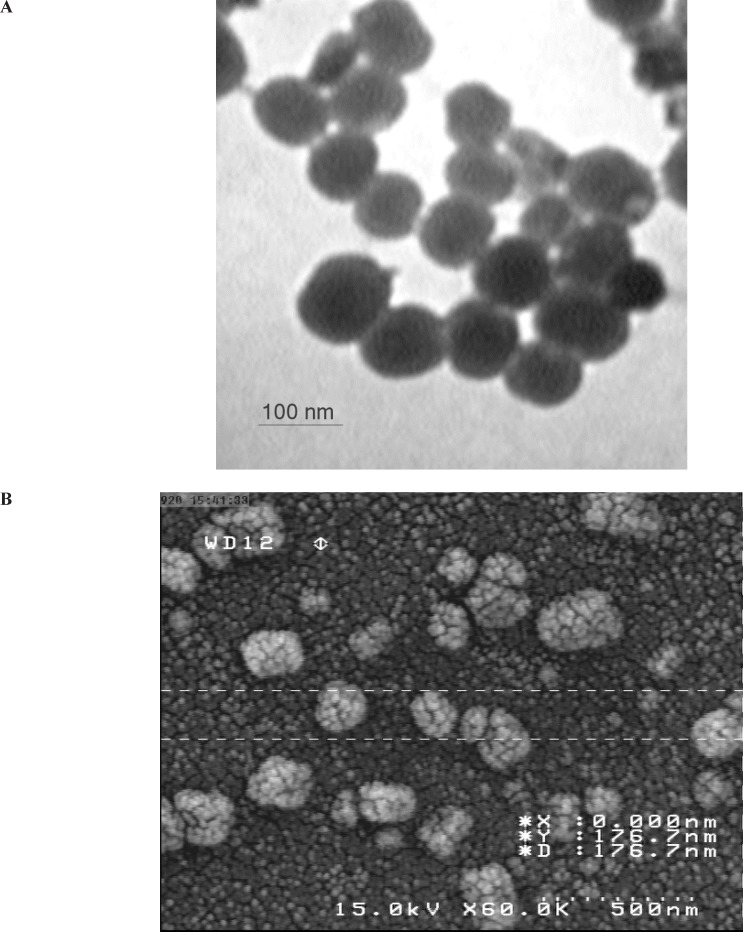
Transmission electron (A) and scanning electron (B) microscopy study of optimized nanoparticles

The shape and surface texture of the nanoparticles could be detected using a number of sophisticated techniques such as TEM or SEM, respectively. Nanoparticles showed a round and smooth surface in TEM. The morphology of curcumin loaded nanogels as prepared is shown in Figure 1 (A and B). Nanoparticle size was determined by TEM, which proved its sphericity. The particle size ranged between 150 and 200 nm (Figure 2A). The SEM of Nanoparticles proved their smooth surface texture (Figure 2B).

*The e* *ﬀ* *ects of curcumin and nanocurcumin on inhibition and proliferation of MDA-MB231 cell line*

**Figure 3:**
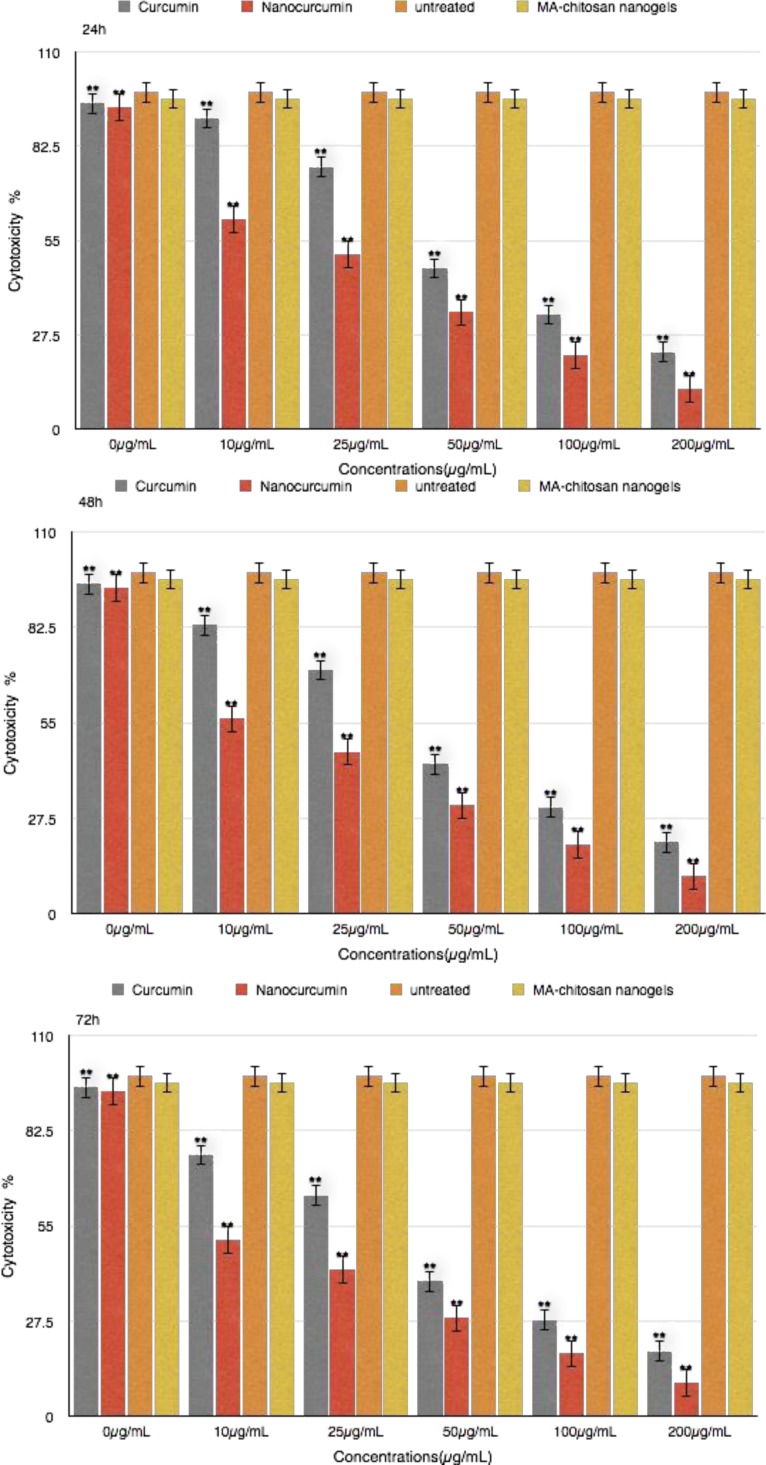
Cytotoxicity effects of curcumin and nanocurcumin on MDA-MB231 cell line

The eﬀect of curcumin and nanocurcumin were studied as a dose-response experiment . Proliferation of MDA-MB231 cells was significantly inhibited by curcumin and nanocurcumin in a concentration-dependent manner. (P<0.01) (Figure 3)

The 50% inhibition concentration (IC50) values curcumin and nano curcumin on MDA-MB231 cells were determined. IC50 was determined by probit analysis using the Pharm PCS (Pharmacologic Calculation System) statistical package (Springer- Verlag, USA). The IC50 curcumin after 24 h , 48 h and 72 h for MDA-MB231 cell line was 79.58μg/mL and 53.18μg/mL and 30.78μg/mL whereas this value for nanocurcumin was 37.75μg/mL and 23.25μg/h and 12.99μg/mL, respectively (P<0.01) .There were significant differences in IC50 curcumin and nanocurcumin (P<0.01).

## Discussion

Cancer drug resistance has been reported to develop following treatment. Therefore, there is need to explore into new anti-cancer drugs. Medicinal plants have been used as a traditional treatment agent for numerous human diseases since ages in many parts of the world. In rural areas of the developing countries, they continue to be used as the primary source of medicine. About 80 % of the people in developing countries use traditional medicines for their health care. The frequent use and misuse of the currently used therapeutic agents has led to the evolution of resistant strains of common pathogens as well as increased incidence of adverse effects associated with their usage. Hence, the search for alternative products continues, and natural phytochemicals isolated from plants used as traditional medicines are considered as a good alternative source. As only 1 % of approximately 5,00,000 plant species worldwide has been phytochemically investigated until date, there is great potential for discovering novel bioactive compounds. Turmeric , a rhizome of ***Curcuma longa***, is a flavourful yellow-orange spice. Its plant is 3 feet in height and has lance-shaped leaves and spikes of yellow flowers that grow in a fleshy rhizome or in underground stem. An orange pulp contained inside the rhizome constitutes the source of turmeric medicinal powder (14). Components of tumeric are named curcuminoids, which include mainly curcumin (diferuloyl methane), demethoxycurcumin, and bisdemethoxycurcumin. Curcumin (diferuloylmethane) is a polyphenol derived from ***Curcuma longa*** plant, commonly known as turmeric. The active constituents of turmeric are the flavonoid curcumin (diferuloylmethane) and various volatile oils including tumerone, atlantone, and zingiberone. Other constituents include sugars, proteins, and resins. The best-researched active constituent is curcumin, which comprises 0.3-5.4% of raw turmeric. Curcumin has been used extensively in ayurvedic medicine for centuries, as it is nontoxic and has a variety of therapeutic properties including antioxidant, analgesic, anti-inflammatory, antiseptic activity, and anticarcinogenic activity (15). Curcumin, being a lipophilic molecule, interacts with the cellular membrane and is subsequently internalized, probably by diffusion. The higher curcumin uptake by tumor cells, against normal ones, has been assigned to various hypothetic factors, including the different mem- brane structure, protein composition and larger size (16). The antioxidant activity of curcumin has been identified as the key mechanism by which this dietary phytochemical prevents cancer *in vivo*. Curcumin also allows reduction of oxidative and inflammatory stress in Alzheimer patients (17). Indeed, curcumin has also been shown to inhibit mediators of inflammation such as cyclo-oxygenase-2 and lipooxygenase (two enzymes involved in inflammation), inducible nitric oxide synthase (enzyme that catalyzes the production of nitric oxide), cytokines and NFKB (18,19). As a natural product, curcumin is nontoxic and has diversified effects in various oral diseases. About 40-85% of an oral dose of curcumin passes through the gastrointestinal tract unchanged, with most of the absorbed flavonoid being metabolized in the intestinal mucosa and liver. Due to its low rate of absorption, curcumin is often formulated with bromelain for increased absorption and enhanced anti-inflammatory effect (14).

Bioavailability of a drug to the cells, whether *in vitro *or *in vivo*, is critical for its optimal efficacy. Agents which affect the metabolism of drugs, liposomal encpasulation or nanocapsulation methods are employed to enhance the bioavailability. To enhance the solubility of drugs in aqueous solvents, increase their bioavailability, enhance serum half-life, for tumor cell targeting and bioimaging, nanotechnology has recently emerged as a new technology of choice. Conjugations of drugs with lipids, carbohydrates, and proteins have been used to make nanoparticles (20).

Nanotechnology and nanomedicine offer potentials for development of nano sized delivery systems for curcumin. Shaikh *et al. *developed nanoparticles encapsulating curcumin prepared by the emulsion technique. The *in vivo *pharmacokinetics revealed that nanoparticles-incorportaed curcumin achieved a 9-fold increase in oral bioavailability as compared to curcumin administered with piperine as absorption enhancer (21). Tsai *et al. *prepared an optimized polylactic-co-glycolic acid (PLGA) nano-formulation of curcumin which resulted in a 22-fold higher oral bioavailability in rats as compared to conventional curcumin (22). Curcumin loaded dextran sulphate-chitosan nanoparticles showed preferential killing of cancer cells compared to normal cells, indicating potential in targeting (23).

A very promising delivery system appears to be “nanocurcumin”, polymeric nanoparticleencapsulated curcumin, readily dispersed in aqueous media and with confirmed anti-cancer potentials in preclinical *in vivo *models. Nanocurcumin retained the mechanistic specificity of free curcumin, inhibiting the activation of the seminal transcription factor NF-*κ*B and reducing steady state levels of pro-inflammatory cytokines like ILs and TNF-α (24). Nanocurcumin developed by Bhawana *et al. *and prepared by wet-milling technique, in size range of 2–40 nm was shown to express stronger antimicrobial potential. It remains to be seen whether the same nanocurcumin will display enhanced anti-cancer activity as well (25). A similar approach in reducing the size of curcumin crystals was proposed by Gao *et al. *to produce nanosupsensions for intravenous delivery (26).

Wu *et al. *developed water-dispersible hybrid nanogels for intracellular delivery of curcumin aiming at photodermal therapy (27). The hybrid combines optical label (Au/Ag bimetallic nanoparticle, polystyrene gel layer, polyethylene gel and provides potent cytotoxicity against B16F10 cells by combined chemo-phototermal treatment. Anti-inflammatory activity of curcumin was also found to be enhanced through delivery via o/w nanoemulsions, as evaluated in mouse ear inflammation model. In comparison, Tween-based formulations failed to achieve the same effect (28). Gupta et al recently reported chitosan polymers blended non-covalently to encapsulate curcumin using the capillary-microdot technique (29). These curcumin nanoparticles showed higher efficacy against breast cancer cells and had the potential to treat breast tumors by local, sustained, and long-term therapeutic delivery as a biodegradable system (30).

FTIR spectra of the formed nanogels were studied. The structure of MA-chitosan nanogels (ma-ch), Myristic acid (m) and chitosan (k) characterized with FT-IR spectrometry are shown in Figure1. Following the covalent combination between Chitosan and Myristate to make MA-Chitosan, the absorption band of N-H bending of primary amines at 1632cm^−1 ^of Chitosan and 1700cm^−1^ of C = O stretch of Myristate are moved to a higher position in 1638cm^−1^ in MCS (Figure 1). This could be related to produce amid groups between Myristate and Chitosan. The N – H and O – H stretching vibration bands are overlapping at a strong peak at 3433cm^−1^ in Chitosan and a sharp and high energy at 3438cm^−1^ in MA-Chitosan structure (Figure1.ma-ch). This could be related to the hydrogen bonds between – OH and – NH groups. Similar results have been reported by Mitra *et al* (31).

In the present investigation, curcumin encapsulated chitosan nanoparticles were prepared successfully. A physical evaluation and electron microscope screening supported the suitability for intranasal administration (Figure 2) .We prepared curcumin-loaded nanogels to enhance solubility and investigate their ability to inhibit the proliferation of breast cancer cells in vitro. When examined for its ability to suppress the growth of cancer cells, we found that curcumin -loaded nanogels was at least twice as potent as free curcumin, possibly due to enhanced uptake. It comprised investigation into extended treatment duration and observation on molecular processes. 

The initial stage involved study on breast cancer cell proliferation and exposure to wide range of curcumin and nanocurcumin concentrations. Different concentrations of curcumin and nanocurcumin at 24 h, 48 h and 72 h had different cytotoxicity effects on MDA-MB231 cell line. The proliferation of MDA-MB231 cell line was regulated by dose of curcumin in overall 50 % cell death occured with 53.18 μg/mL and 23.25 μg/mL with 48 h treatment for curcumin and nanocurcumin respectively.  

There was a significant difference between curcumin and nanocucumin effects on growth depression of MDA-MB231cells (P<0.01).It could be concluded that these nanogels are effective at low dose rates, with controlled and effective release and could be applied as a anticancer strategy. The chitosan myristic acid nanogels are effective at low dose rates, with controlled and effective release and could be applied as an anticancer strategy (32).
